# The American Text Book of Prosthetic Dentistry

**Published:** 1897-05

**Authors:** 


### Bibliographical.

THE AMERICAN TEXT BOOK OF PROSTHETIC DENTISTRY. In Contributions by Eminent Authorities Edited by Charles J. Essig, M. D., D. D. S., Professor of Mechanical Dentistry and Metallurgy, Department of Dentistry, University of Penna., Phila. Illustrated with 983 engravings. Publishers: Lea Brothers & Co., Philadelphia and New York.

The contributors of this excellent work on prosthetic dentistry are Drs. H. H. Burchard, Chas. J. Essig, W. W. Evans, C. L. Goddard, G. Molyneaux, R. Ottolengui, A. Tees, Jr, and A. H. Howard. It is worthily dedicated to Dr. Louis Jack.

Its contents comprise twenty-one chapters devoted to the equipment and arrangement of the dental laboratory; metals and alloys used in prosthetic dentistry; principles of metal work; moulding and carving of porcelain teeth; preparation of the mouth, materials and type of dentures; taking impressions; models; dies, counter-dies and moulding; swaged metallic plates; the "bite"; selecting and arranging teeth; attachment to plate, and finishing; English tube teeth; continuous gum; aluminum and fusible alloys; vulcanite; celluloid and zylonite; temperaments and their characteristics of the teeth; artificial crowns, bridge-work; hygienic relations and care of artificial dentures; and palatal mechanism; all of which form a very complete treatise on prosthetic dentistry. Great credit is due Dr. Essig for his energy and labors in presenting to the dental profession such a valuable standard work on prosthetic dentistry, and also to the publishers upon the excellent manner in which it is printed and arranged. The illustrations demand notice not only on account of their number, but also for their excellence. The concluding chapter but one, of this work, by Dr Essig on "Hygienic Relations and Care of Artificial Teeth," is well worthy of perusal.

